# Renal Pelvis Opacification on Postmyelography Computed Tomography as an Indicator for Cerebrospinal Fluid Loss in Spontaneous Intracranial Hypotension

**DOI:** 10.1007/s00062-021-01042-0

**Published:** 2021-06-25

**Authors:** Eike I. Piechowiak, Laura Bär, Levin Häni, Mattia Branca, Johannes Kaesmacher, Pasquale Mordasini, Andreas Raabe, Christian T. Ulrich, Jan Gralla, Jürgen Beck, Tomas Dobrocky

**Affiliations:** 1 Department of Diagnostic and Interventional Neuroradiology, Inselspital, Bern University Hospital, and University of Bern, Freiburgstrasse 8, 3010 Bern, Switzerland; 2 Department of Neurosurgery, Inselspital, Bern University Hospital, and University of Bern, Bern, Switzerland; 3 CTU Bern, Institute of Social and Preventive Medicine, University of Bern, Bern, Switzerland; 4 Department of Interventional, Pediatric and Diagnostic Radiology, Inselspital, University Hospital, and University of Bern, Bern, Switzerland; 5 Department of Neurosurgery, Medical Center, University of Freiburg, Freiburg, Germany

**Keywords:** CSF leak, CSF venous fistula, Spine, Orthostatic headache, CSF resorption

## Abstract

**Purpose:**

To assess early renal pelvis opacification on postmyelography computed tomography (CT) as a marker for cerebrospinal fluid (CSF) loss in patients with spontaneous intracranial hypotension (SIH).

**Methods:**

The SIH patients referred to our hospital between January 2012 and May 2018 were retrospectively reviewed and divided into 2 groups based on the presence of spinal longitudinal extrathecal CSF collection (SLEC): (1) SLEC(+) with, and (2) SLEC(−) without proof of SLEC on multimodal imaging. Non-SIH patients (*n* = 20) undergoing CT myelography served as controls. The renal pelvis density on postmyelography CT was measured in all patients. Mean difference in renal pelvis density between the groups was calculated.

**Results:**

In total, 111 SIH patients (mean age 48 ± 13 years; 60% female) were included, 71 (64%) SLEC(+) and 40 (36%) SLEC(−). The adjusted renal pelvis density in the SLEC(+), SLEC(−), and the non-SIH group was 108 Hounsfield unit (HU), 83 HU, and 32 HU, respectively, resulting in a significant difference between SLEC(+) vs. control group 1 (75 HU, *p* < 0.001), SLEC(−) vs. control group 1 (50 HU, *p* < 0.001), and a tendency for higher density in SLEC(+) than SLEC(−) (25 HU, *p* = 0.16).

**Conclusion:**

Increased renal pelvis opacification on postmyelography CT was observed in SIH patients, even in the absence of a CSF leak or a CSF venous fistula, when compared to non-SIH patients. Although the provenance of early renal opacification in SLEC (−) SIH patients remains unclear, our results suggest that it may be a surrogate for increased spinal CSF resorption via spinal arachnoid granulations and along spinal nerve sheaths occult to direct imaging.

## Introduction

Spontaneous intracranial hypotension (SIH) has an estimated incidence of 2–5 per 100,000 inhabitants and is usually caused by a spinal cerebrospinal fluid (CSF) leak [1]. SIH patients typically present with orthostatic headache, but other symptoms, such as nausea, neck stiffness, and hearing alterations have been reported [2]. Women are twice as likely to be affected as men and the mean age at presentation is around 40 years [1, 3].

Several underlying pathomechanisms may lead to SIH. First, in patients with a spinal longitudinal extradural CSF collection (SLEC(+)), a calcified disc extrusion, an osteophyte causing a dural breach, or a leaking spinal nerve root cyst may be demonstrated [4, 5]. Second, in patients without a SLEC (SLEC(−)), a CSF venous fistula (CSFVF)—a communication between the subarachnoid space (SAS) and the venous system—has been proposed, and may be demonstrated as a hyperdense paraspinal vein sign on computed tomography (CT) myelography [6, 7]. Depending on the diagnostic technique used and the inclusion criteria applied, the frequency of spinal CSFVF in SIH without SLEC may vary between 15% and 75% [8]. Finally, in some patients with typical orthostatic headache who demonstrate characteristic findings on brain magnetic resonance imaging (MRI), multimodal spine imaging including non-enhanced MRI, intrathecal gadolinium-enhanced MRI, CT myelogram, or conventional myelogram may fail to reveal the underlying pathology. It is still unclear whether these represent false-negative spine imaging findings, especially given the challenging diagnosis of a CSFVF, or alternative pathomechanisms, such as hypercompliance of the thecal sac, increased CSF resorption, or decreased CSF secretion should be considered [1].

The primary pathway of CSF resorption may be via intracranial arachnoid granulations (Pacchionian granulations or bodies). In addition, CSF resorption through spinal arachnoid villi [9], and direct lymphatic drainage into deep cervical and prevertebral lymph nodes via epidural lymphatics have been reported [10]. These pathways of CSF circulation, however, are difficult to directly visualize on routine clinical imaging, and we speculate as to whether renal pelvis opacification might be a valuable marker thereof.

The main goal of our study was to compare renal pelvis opacification on postmyelography CT (PMCT) as a marker of CSF loss or CSF hyperresorption between patients with SIH and non-SIH patients.

## Methods

Institutional review board approval was obtained and, due to the retrospective nature of the study, the need for informed consent was waived. The registry was approved by the local ethics committee. All consecutive patients referred to our hospital between January 2012 and May 2018 with suspected SIH were retrospectively evaluated. Only the diagnostic studies performed before treatment were considered. SIH was defined according to the international classification of headache disorders (ICHD-3/7.2.3: positive spinal or cerebral imaging or low CSF pressure of < 60 mm H_2_O, especially orthostatic headache in temporal relation to the low CSF pressure or CSF leakage, no known trauma or dural puncture).

Most of the patients had been included in previous studies investigating different parameters, including optic nerve sheath ultrasonography, surgical dural closure, CSF dynamics, dynamic CT myelography, and brain MRI [5, 11–15].

### Subjects

**Fig. 1:**
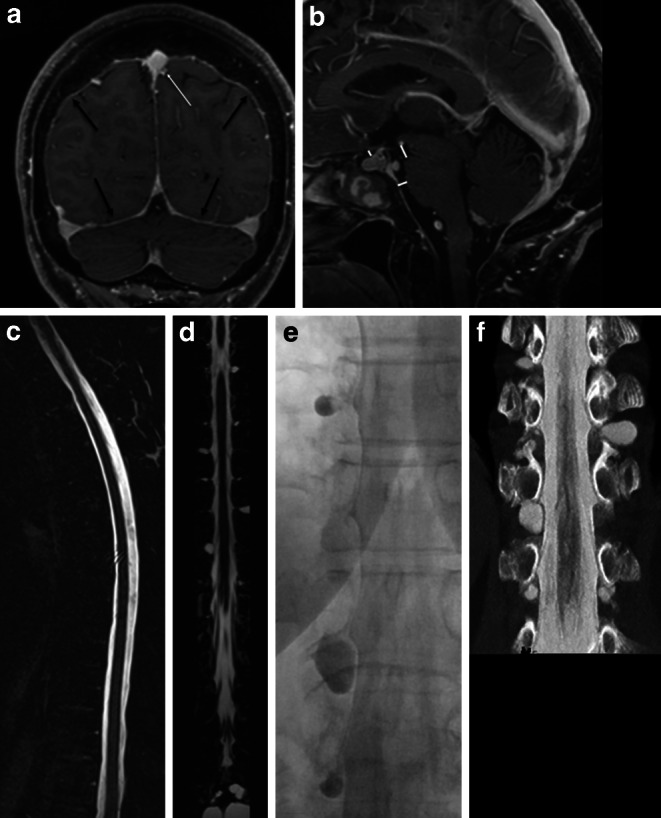
Patient with orthostatic headache. **a**, **b** Brain MRI demonstrating pachymeningeal enhancement (**a**; *black arrows*), venous engorgement (**a**; *white arrow*), no subdural collection, effaced suprasellar (< 4.0 mm) and prepontine (< 5.0 mm) cistern, and decreased mamillopontine distance (< 6.5 mm); SIH score = 8 indicating high likelihood of SIH. **c**, **d** Spine MRI does not show a spinal longitudinal extradural CSF collection; however, multiple spinal nerve root cysts are demonstrated. **e**, **f** Conventional dynamic myelography and postmyelography CT demonstrate filling of the nerve root cysts without epidural contrast agent leakage or a CSF venous fistula. *CSF* cerebrospinal fluid, *CT* computed tomography, *MRI* magnetic resonance imaging, *SIH* spontaneous intracranial hypotension

**Fig. 2:**
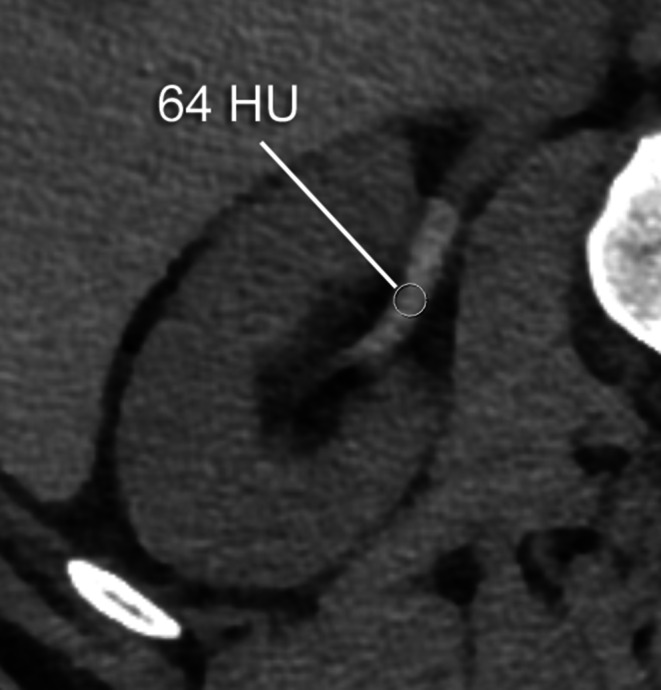
Transversal PMCT after previous intrathecal contrast agent application in a 55-year-old patient with orthostatic headache without epidural CSF collection (SLEC(−)) demonstrating opacification of the renal pelvis and a density measurement using a circular ROI with a mean of 64 Hounsfield units. *CSF* cerebrospinal fluid, *PMCT* postmyelography computed tomography,* ROI* region of interest

**Fig. 3:**
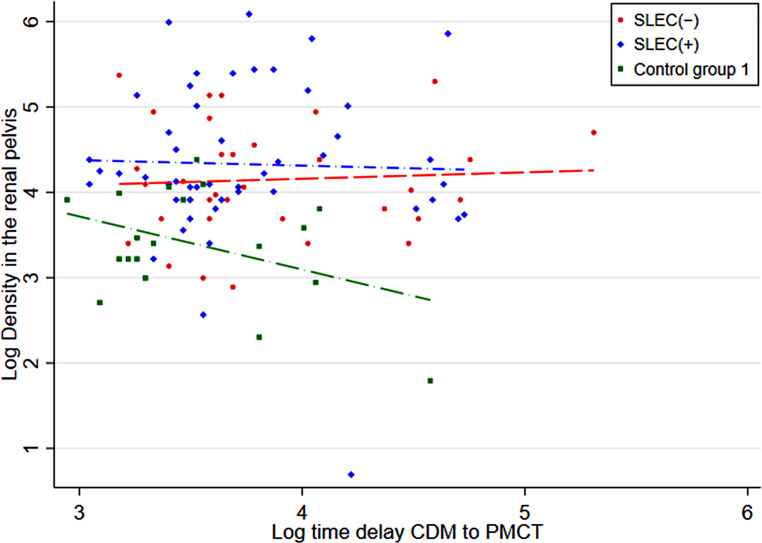
Scatter plot with logarithmic axes demonstrating the renal pelvis density and the time delay between intrathecal contrast agent application on conventional dynamic myelography (CDM) and postmyelography CT (PMCT). (1) SLEC(−): patients without a longitudinal extrathecal CSF collection; (2) SLEC(+): with a longitudinal extrathecal CSF collection; (3): control group 1 including non-SIH patients in whom CT myelography was performed to rule out spinal cord or nerve root compression. *CSF* cerebrospinal fluid, *CT* computed tomography

Patients were divided into 4 groups: (1) SLEC(+): patients with SIH and SLEC confirmed on multimodal imaging including spine MRI, conventional dynamic myelography (CDM) and PMCT (Fig. 1), (2) SLEC(−): SIH patients without SLEC on multimodal spine imaging (Fig. 1), (3) control group 1: consecutive patients seen during a 3-month period, without orthostatic headache, in whom CT myelography was performed to rule out spinal cord or nerve root compression, (4) control group 2: patients without orthostatic headache who underwent unenhanced CT of the spine for unrelated indications and were used as a reference standard for the study evaluation but not further evaluated.

Exclusion criteria for both control groups were malignant disease or impaired glomerular filtration rate (GFR < 60 ml/min). Additional exclusion criteria for SIH patients were any history of trauma, spinal intervention, or lumbar puncture.

### Procedure

All patients admitted to our hospital with a clinical suspicion of SIH were evaluated according to our standard protocol. First, a detailed medical history was obtained, and a physical examination was performed by a neurosurgeon. In most patients the leading symptom was orthostatic headache, thus brain MRI was performed to rule out any underlying intracranial pathology.

For spinal imaging, unenhanced MRI including fat-suppressed T2-weighted isovoxel sequences, and prior to 2019 intrathecal gadolinium-enhanced MRI, were acquired first (usually > 48 h before CDM, range 24–144 h). Then, CDM was performed on a monoplane angiosuite (Artis zee multipurpose, Siemens, Erlangen, Germany) by injecting 20 mL Iopamiro 300 (iopamidol, Bracco, Candempino, Switzerland) intrathecally for opacification. Patient positioning was adapted in accordance with the results of the previous imaging; prone when a ventral leak was suspected or lateral decubitus when a spinal nerve root cyst was the presumed source of leakage. The level at which the contrast agent exited the intrathecal compartment and started opacifying the epidural space was considered to be the site of dural breach. The patient was immediately transferred to the CT imaging suite and a PMCT was performed (SOMATOM Definition Edge, Siemens) to identify the possible cause at the level of dural dehiscence, or a CSFVF. A PMCT was performed at the level of CSF leakage and extended to the entire spine only if no contrast leakage was identified on CDM. If no epidural contrast agent was visible in the first PMCT a late-phase PMCT was performed, in general 4–12 h after the initial intrathecal injection, to exclude low-flow leaks.

Opening pressure was recorded after lumbar puncture in lateral decubitus position in all cases.

### Image Analysis

All PMCT images were assessed by 3 readers (L.B., T.D., and E.I.P.) with 1, 8, and 11 years of experience, respectively. The readers were blinded to all other imaging studies and clinical presentation. Structured evaluation was performed on a PACS station (R11.4.1, 2009; Philips, Best, The Netherlands; Sectra, Linkoping, Sweden). Conflicts between readers were resolved by consensus after a joint case discussion.

In patients who had undergone more than one CDM, the PMCT covering the largest part of the spine was analyzed. If multiple CDMs were performed there was a time delay of at least 24 h and no treatment was initiated in between. The time from injection of intrathecal contrast agent (20 mL Iopamiro 300) to early and delayed PMCT (when performed) was recorded. The reviewers reported the presence of the hyperdense paraspinal vein sign. This was rated positive when a tubular/curvilinear opacified structure extending from the thecal sac or from a nerve root sleeve into the paravertebral space was present. The presence of diffuse contrast agent leakage beyond the epidural space into paravertebral soft tissue was also recorded.

In all patients the density in the renal pelvis, the vena cava (either inferior or superior), and the aorta was measured in Hounsfield units (HU) by one reader (L.B.), on 1‑mm slices with soft tissue kernel in the axial plane using a circular region of interest tool (Fig. 2). In patients with delayed images, the measurements were repeated. In all patients, the GFR (in ml/min) was estimated. The findings were reported in a standardized spreadsheet.

### Statistical Analysis

Statistical analysis was carried out using Stata (StataCorp. 2017. Stata Statistical Software: Release 15. College Station, TX, USA: StataCorp LLC). Descriptive analysis was performed using frequencies and percentages for categorical variables and mean (± SD) or median (interquartile range, IQR) for continuous variables. The χ^2^-test and t‑test were used to compare categorical and continuous variables, respectively. The normal distribution of continuous variables was checked and where necessary a log-transformation was applied.

A comparison was made to test for differences in the renal pelvis density and the time from contrast agent injection to PMCT, and to late-phase CT between the SLEC(+), SLEC(−), and control group 1. First, a general Kruskal-Wallis test was applied to test for difference between groups, and an ad hoc unpaired t‑test was used to make a pairwise comparison of the mean of the groups (with Welch’s approximation). When considering the post hoc paired comparisons, the significance level was adjusted using the Bonferroni correction. In addition, a scatterplot to graphically depict the difference between groups was provided.

Furthermore, a comparison between SIH patients (SLEC(+) and SLEC(−)), and control group 1 (non-SIH patients with CT myelography) was performed to test for differences in the density in the renal pelvis, vena cava, and aorta. The method used to compare the groups was a multivariable linear regression with adjustment for eGFR and the time from contrast agent injection to PMCT using robust standard errors to account for the large values present in the data.

## Results

### Demographic and Baseline Characteristics

In total, 111 SIH patients matching the inclusion criteria were investigated at our center during the study period; 71 (64%) SLEC(+) patients with a spinal CSF leak and 40 (36%) SLEC(−) patients without proof of a CSF leak on multimodal spinal imaging (Table 1). In addition, 20 patients investigated with CT myelography to rule out spinal cord or nerve root compression served as control group 1, and 21 patients who underwent an unenhanced CT scan of the lumbar spine (control group 2) served as reference but were not further evaluated in the absence of statistical abnormalities.

**Table 1:** Baseline demographics and characteristics

	SLEC(+) group	SLEC(−) group	Control group 1	*p*-value
Number	71	40	20	–
Age (years) (mean)	45 (± 11)	54 (± 17)	46 (± 8)	0.004
Sex (female)	48 (68%)	19 (47%)	11 (55%)	0.14
eGFR (ml/min)	86 (±7)	81 (±13)	87 (±7)	0.006
*Position in CT*				
Supine	42 (59%)	31 (78%)	–	0.06
Prone	27 (38%)	8 (20%)	–	0.06
Lateral	2 (3%)	1 (3%)	–	1
*Leak location*				
Ventral	49 (69%)	–	–	–
Dorsal	3 (4%)	–	–	–
Lateral	17 (24%)	–	–	–
Unclear	2 (3%)	–	–	–
*Etiology*				
Spinal meningeal diverticula	13 (18%)	–	–	–
Microspur	50 (71%)	–	–	–
Unclear	8 (11%)	–	–	–
*Time CDM to PMCT (min)*	45 (± 23)	61 (± 61)	37 (± 19)	0.04
*Spinal meningeal diverticula*				
None	38 (54%)	13 (33%)	16 (80%)	0.002
1–5	25 (35%)	13 (33%)	2 (10%)	0.09
6–10	7 (10%)	8 (20%)	2 (10%)	0.28
> 10	1 (1%)	6 (15%)	0 (0%)	0.01

*CT* computed tomography, *eGFR* glomerular filtration rate (ml/min), *CDM to PMCT* conventional dynamic myelography to postmyelography CT

The mean age (including control groups) was 48 (± 13) years (range 26–90 years), lower in the SLEC(+) than in the SLEC(−) group (45 vs. 54 years; *p* = 0.004). There was a slight predominance of female SIH patients (67/111, 60%) (Table 1).

All spinal CSF leaks were located between C5 and L4, most frequently in the upper thoracic spine. The underlying pathology in SLEC(+) patients was a microspur (50/71, 71%) or a leaking spinal nerve root cyst (13/71, 18%). In the remaining patients (8/71, 11%) a ventral leak without proof of a penetrating microspur on PMCT was responsible for CSF loss (e.g. non-calcified spur or resorbed microspur). The mean opening pressure on lumbar puncture in SLEC(+) and SLEC(−) patients was 8.9 cm H_2_O (range 0–18 cm H_2_O) and 12.3 cm H_2_O (range 3–209 cm H_2_O), respectively, with 23 SLEC(+) (31%) and 2 SLEC(−) patients (4%) having an opening pressure of < 6 cm H_2_O.

There was a significant difference in time delay between intrathecal contrast agent injection and PMCT between SLEC(−) and control group 1 (61 min vs. 37 min; *p* = 0.007). A tendency for a longer delay between SLEC(+) vs. control group 1 (45 min vs. 37 min; *p* = 0.06) and SLEC(+) vs. the SLEC(−) group (61 min vs. 45 min; *p* = 0.13) was noted. There was no significant difference in time delay between intrathecal contrast agent injection and late-phase PMCT between SLEC(+) and SLEC(−) patients (249 min vs. 245 min; *p* = 0.90). A significant between-group difference in renal function (ml/min) was recorded: SLEC(+): 86; SLEC(−): 81; control group 1: 87; control group 2: 87 (*p* = 0.006).

### Imaging Features

The PMCT was performed with the patient in the supine position in 73 cases (66%), prone in 35 (32%), and lateral decubitus in 3 cases (3%). A PMCT scan of the entire spine was obtained in 31 patients (44%) in the SLEC(+) and 32 patients (80%) in the SLEC(−) group. In the remaining patients, only the level harboring the leak was scanned (mostly thoracic). A late-phase PMCT was available for 26 patients. In 26 (37%) SLEC(+) patients, a diffuse leakage of contrast agent through the neuroforamen beyond the epidural space into paravertebral tissue was observed. In 2 (1.5%) SLEC(+) patients, the hyperdense paraspinal vein sign was demonstrated, one originating at T12/L1, and one at C7/T1. Renal pelvis density could not be measured in 3 SLEC(−) and in 21 SLEC(+) patients since it was not included in the scan volume. Renal pelvis density was adjusted for the time interval between contrast agent injection and PMCT, and eGFR.

A scatter plot showing the time delay from intrathecal contrast agent application on CDM to PMCT versus the renal pelvis density is shown in Fig. 3. The adjusted renal pelvis density in the SLEC(+) and SLEC(−) groups and in control group 1 was 108 HU (range 13–500 HU), 83 HU (range 18–215 HU), and 32 HU (range 3–35 HU), respectively. This resulted in a significant difference in adjusted renal pelvis density between SLEC(+) vs. control group 1 (75 HU, *p* < 0.001), SLEC(−) vs. control group 1 (50 HU, *p* < 0.001), and a tendency for higher density in SLEC(+) vs. SLEC(−) (25 HU, *p* = 0.16) (Table 2).

**Table 2:** Difference between groups in mean renal pelvis density measured in Hounsfield units

	Density in the renal pelvis	
Comparison	Difference (95% confidence interval)	*p*-value
SLEC(+) group vs. control group 1	75.0 (44.9; 105.1)	< 0.001
SLEC(−) group vs. control group 1	50.3 (27.1; 73.4)	< 0.001
SLEC(+) group vs. SLEC(−) group	24.7 (−9.6; 59.0)	0.16

No difference in the density in the aorta or the vena cava was observed between groups. For reference, the renal pelvis density in control group 2 with unenhanced spine CT was 8 HU (range 3–35 HU). No further relevant results were obtained with respect to control group 2 and are therefore not discussed further.

## Discussion

Our results demonstrate increased renal pelvis opacification on PMCT in SIH patients, even in the absence of a CSF leak or a CSFVF, when compared to non-SIH patients. SLEC(+) patients demonstrated the highest renal pelvis opacification on PMCT when adjusted for time delay between intrathecal contrast agent application and PMCT, and for eGFR (*p* < 0.001).

An important factor accounted for by our study is that results were corrected for time between contrast injection and the PMCT, as well as GFR, which both have an influence on the renal excretion of contrast agent.

A study by Kinsman et al. on early renal pelvic opacification on CT myelography concluded that opacification was more common in patients with confirmed or suspected CSFVF than in those with dural leaks [16]. In contrast to their study, which evaluated renal pelvis opacification qualitatively (yes/no), we quantified the opacification in order to evaluate differences between groups. This demonstrated increased renal pelvis density in patients undergoing CT myelography for non-SIH indications, as compared to unenhanced scans (control group 2), which supports the hypothesis of immediate resorption of intrathecal contrast agent in all study participants, although at a lower rate than in SIH patients.

**Fig. 4:**
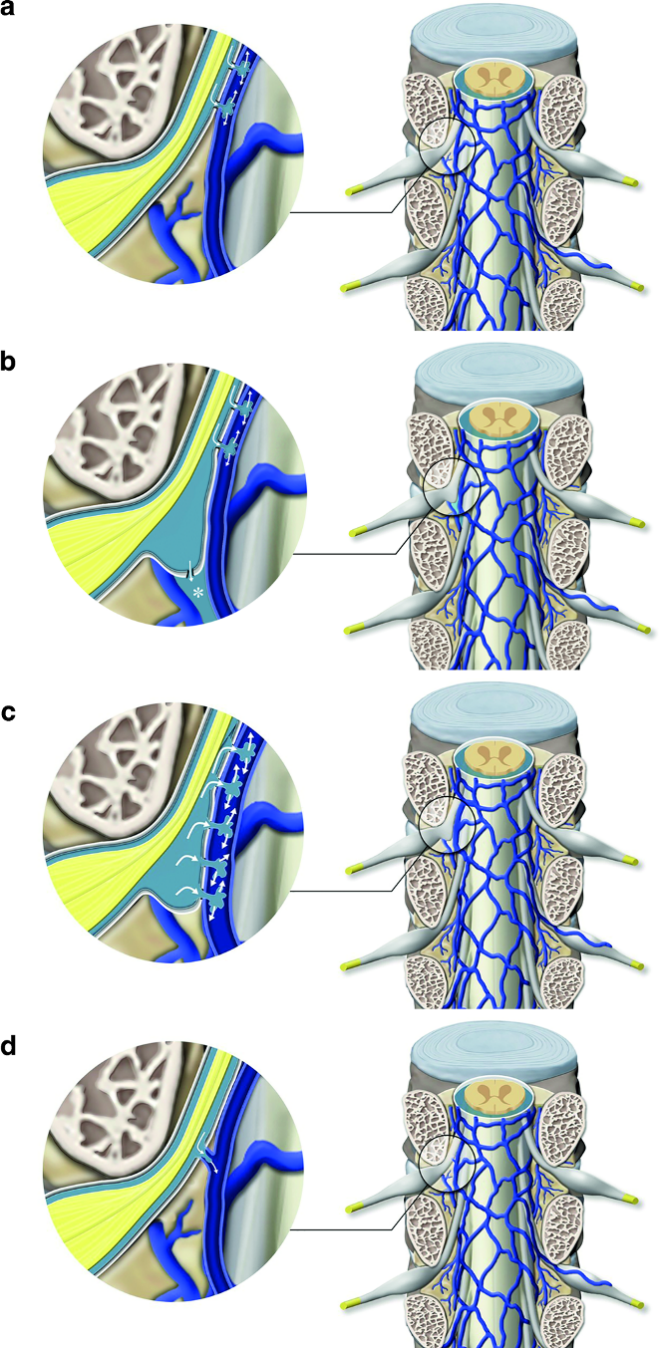
Illustration of the spine depicting the epidural venous plexus (*blue*). Spinal arachnoid granulations (SAG) are illustrated with the outflow (*arrow*) of cerebrospinal fluid (CSF) into the adjacent radicular vein. **a** Illustration of the normal CSF resorption along spinal arachnoid granulations. **b** Illustration of a spinal CSF leak. CSF leaks from the intrathecal to the epidural compartment via a dural breach (*asterisk*) where it is resorbed and finally excreted into the renal collecting system. In addition, resorption through SAGs is demonstrated. **c** Illustration of a spinal meningeal cyst with increased CSF resorption into the adjacent epidural vein via SAGs. This might be the underlying pathomechanism of CSF hyperresorption in SIH. Depending on the amount of contrast agent outflow through the SAG the finding may remain occult on imaging; or may be demonstrated as a CSF venous fistula (CSFVF) in a case of high flow. **d** Illustration of a CSFVF which has formed as a de novo abnormal connection

What do we know about CSF resorption? Three important pathways of CSF absorption may be distinguished: first, the widely recognized route via cranial arachnoid granulations [9]; second, via spinal arachnoid granulations (SAG), which are mostly adjacent to a radicular vein, and mainly located on the dorsal nerve root (Fig. 4a; [17, 18]); third, and less well known, along cranial and spinal nerve sheaths into the lymphatic system, which then drain into lymph nodes [19]. Direct lymphatic drainage into deep cervical and prevertebral lymph nodes via epidural lymphatics, which originate from the spinal meninges and are well developed in the upper spine, has been reported by Miura et al. [10]. In humans, spinal CSF absorption accounts for approximately 20% of the total outflow [20].

Besides the resorption via SAGs, contrast agent that has leaked into the epidural space through a dural breach might additionally be resorbed from this compartment and finally excreted into the renal collecting system (Fig. 4b). In SLEC(+) patients, diffuse leakage of contrast agent beyond the epidural space into paravertebral tissue, indicating a high-flow leak, was associated with a significantly higher renal pelvis density in our study (*p* < 0.001). This finding supports the assumption of an additional, and important pathway of CSF resorption from the epidural space. Our results are in line with a recent study by Behbahani et al. reporting on early renal opacification in patients with and without a dural tear [21]. With a smaller study population, and also including cases without SIH according to the ICHD‑3 criteria, the group reported an increased renal pelvis density in patients with CSF leak but not in patients without.

On the other hand, in SLEC(−) patients without proof of a CSF leak or a CSFVF, the underlying pathomechanism leading to increased renal pelvis opacification on PMCT remains unclear. Whether these patients should be considered false negatives for a dural CSF leak, or a CSFVF, or whether other forms of CSF loss may be responsible for this finding remains undetermined. As elucidated above, intrathecally injected contrast agent is absorbed through SAG and spinal nerve sheaths and has been reported to commence almost immediately after lumbar injection [22]; however, these pathways of CSF resorption are difficult to directly visualize on imaging and may be occult for the neuroimager. Thus, increased renal pelvis opacification on PMCT in SLEC(−) patients may be considered an indicator for increased spinal CSF resorption via SAG and spinal nerve sheaths, leading to functional CSF hypotension (Fig. 4c).

A tendency toward higher renal pelvis opacification on PMCT in SLEC(+) compared to SLEC(−) patients was demonstrated, indicating a potentially increased resorption in the former group when adding the resorption from the intrathecal and epidural spaces.

A hyperdense paraspinal vein sign was present in only 2 patients in our population, both with a coexisting spinal CSF leak. None of the SLEC(−) patients had a hyperdense paraspinal vein. In several studies the detection rate for CSFVF varied substantially depending on the imaging modality used and the time from contrast agent injection to PMCT [23–25]. Recently, Schievink et al. reported a high yield for detection of CSFVF on digital subtraction myelography in lateral decubitus position [26]. The explanation for the low detection rate in our population may be partly that this technique was not used in our study and patients without epidural CSF collection had conventional myelography in the prone position. Furthermore, in our experience, differentiating a true CSFVF from diffuse epidural contrast agent spillage may be challenging on PMCT. Whether CSFVF truly represents an epidural vein directly connected to the intrathecal space remains controversial, and the underlying mechanism leading to fistula formation is not well understood (Fig. 4d). Alternatively, CSFVF could represent a physiological increase in CSF drainage via SAG into a paraspinal vein (Fig. 4c); however, to our knowledge, there is no direct evidence that confirms this hypothesis.

Kranz et al. postulated that a focal rupture or failure of the SAG may be responsible for unregulated CSF resorption into an adjacent radicular vein, which could represent a CSFVF [27]. Support for their hypothesis came from an association of a CSFVF with a spinal meningeal diverticulum, which was present in 82% of their patients [23].

The major strength of our study is the comparison of SIH patients, including those with and without a CSF leak. In addition, quantitative measurement of renal pelvis opacification was performed as opposed to simple qualitative measurement, which adds insight into the dynamic concept of SIH. Furthermore, our study provides in-depth discussion and graphical illustrations of CSFVF, which we consider a form of increased CSF resorption as opposed to a single direct connection between the CSF space and the venous system.

The main limitations of our study relate to its retrospective and monocentric nature. Newer techniques for the detection of CSFVF, such as lateral decubitus imaging, were not used at the beginning of the study period, which might have contributed to false negative results for the presence of CSFVF. Additional information on the 21 (of 71) SLEC(+) patients in whom the renal pelvis density could not be measured would have increased the quality of the data.

Although the provenance of early renal opacification in SLEC(−) patients remains unclear, our results suggest that it may be a surrogate for increased spinal CSF resorption.

Future research should include the development of new diagnostic procedures to detect levels of increased spinal CSF resorption and the relation to spinal meningeal diverticula that are not CSFVF.

## Conclusion

Our study demonstrated increased renal pelvis opacification on PMCT in SIH patients, even in the absence of a CSF leak or a CSFVF, when compared to non-SIH patients.

## Abbreviations

CDM: Conventional dynamic myelography
CSF: Cerebrospinal fluid
CSFVF: Cerebrospinal fluid venous fistula
CT: Computed tomography
DCTM: Dynamic CT myelography
GFR: Glomerular filtration rate (ml/min)
HU: Hounsfield unit
PMCT: Postmyelography computed tomography
SAG: Spinal arachnoid granulation
SAS: Subarachnoid space
SIH: Spontaneous intracranial hypotension
SLEC: Spinal longitudinal extradural CSF collection
